# Corrigendum to “In Vitro Evaluation of Probiotic Properties of Lactic Acid Bacteria Isolated from Some Traditionally Fermented Ethiopian Food Products”

**DOI:** 10.1155/2020/6401356

**Published:** 2020-07-24

**Authors:** Guesh Mulaw, Tesfaye Sisay Tessema, Diriba Muleta, Anteneh Tesfaye

**Affiliations:** ^1^Department of Microbial, Cellular and Molecular Biology, College of Natural and Computational Sciences, Addis Ababa University, Addis Ababa 1176, Ethiopia; ^2^Department of Biology, College of Natural and Computational Sciences, Aksum University, Axum 1010, Ethiopia; ^3^Institute of Biotechnology, College of Natural and Computational Sciences, Addis Ababa University, Addis Ababa 1176, Ethiopia

In the article titled “In Vitro Evaluation of Probiotic Properties of Lactic Acid Bacteria Isolated from Some Traditionally Fermented Ethiopian Food Products” [1], the headings for Sections 3.3.4 and 3.3.5 were reversed. The correct headings are as follows:

  3.3.4. Bacterial Adhesion to Stainless Steel Plates
  3.3.5. Antibiotic Susceptibility Test
