# Supplementary material for: In Vitro Evaluation of Probiotic Properties of Lactic Acid Bacteria Isolated from Some Traditionally Fermented Ethiopian Food Products
Source: Int J Microbiol. 2019 Aug 25;2019:7179514. doi: 10.1155/2019/7179514 (PMC6732631; doi:10.1155/2019/7179514)
Supplement: Supplementary Materials — Three (Teff dough, Ergo, and Kocho) traditionally fermented Ethiopian food products were used as the isolation source. The isolates were designated with E for Ergo, T for Teff dough, and K for Kocho, followed with different numbers. In general, the abovementioned traditionally fermented food products are prepared at the household level and their consumption is commonly practiced throughout the country. [file 7179514.f1.pdf]

Number	Isolates code	KOH test	Remark	Catalase test	Remark	Spore staining
1.	T031	-ve		-ve	Passed	-ve
2.	T032	+ve		+ve	Discarded	-
3.	T033	-ve		-ve	Passed	-ve
4.	T034	-ve		-ve	Passed	-ve
5.	T035	-ve		-ve	Passed	-ve
6.	T036	+ve		+ve	Discarded	-
7.	T037	+ve		+ve	Discarded	-
8.	T038	+ve		+ve	Discarded	-
9.	T039	-ve		-ve	Passed	-ve
10.	T040	-ve		-ve	Passed	-ve
11.	T041	-ve		-ve	Passed	-ve
12.	T042	-ve		-ve	Passed	-ve
13.	T043	-ve		-ve	Passed	-ve
14.	T044	+ve		+ve	Discarded	-
15.	T045	+ve		+ve	Discarded	-
16.	T046	+ve		+ve	Discarded	-
17.	T047	-ve		-ve	Passed	-ve
18.	T048	-ve		-ve	Passed	-ve
19.	T049	-ve		-ve	Passed	-ve
20.	T050	-ve		-ve	Passed	-ve
21.	T051	-ve		-ve	Passed	-ve
22.	T052	-ve		-ve	Passed	-ve
23.	T053	-ve		-ve	Passed	-ve
24.	T054	-ve		-ve	Passed	-ve
25.	T055	-ve		-ve	Passed	-ve
26.	T056	+ve		+ve	Discarded	-
27.	T057	-ve		-ve	Passed	-ve
28.	T058	-ve		-ve	Passed	-ve
29.	T059	+ve		+ve	Discarded	-
30.	T060	+ve		+ve	Discarded	-
31.	E031	-ve		-ve	Passed	-ve
32.	E032	-ve		-ve	Passed	-ve
33.	E033	-ve		-ve	Passed	-ve
34.	E034	+ve		+ve	Discarded	-
35.	E035	+ve		+ve	Discarded	-
36.	E036	-ve		-ve	Passed	-ve
37.	E037	-ve		-ve	Passed	-ve
38.	E038	+ve		+ve	Discarded	-
39.	E039	-ve		-ve	Passed	-ve
40.	E040	-ve		-ve	Passed	-ve
41.	E041	+ve		+ve	Discarded	-
42.	E042	+ve		+ve	Discarded	-
43.	E043	+ve		+ve	Discarded	-
44.	E044	+ve		+ve	Discarded	-
45.	E045	-ve		-ve	Passed	-ve
46.	E046	-ve		-ve	Passed	-ve
47.	E047	-ve		-ve	Passed	-ve

48.	E048	-ve		-ve	Passed	-ve
49.	E049	+ve		+ve	Discarded	-
50.	E050	-ve		-ve	Passed	-ve
51.	E051	-ve		-ve	Passed	-ve
52.	E052	-ve		-ve	Passed	-ve
53.	E053	-ve		-ve	Passed	-ve
54.	E054	+ve		+ve	Discarded	-
55.	E055	-ve		-ve	Passed	-
56.	E056	+ve		+ve	Discarded	-
57.	E057	-ve		-ve	Passed	-ve
58.	E058	-ve		-ve	Passed	-ve
59.	E059	+ve		+ve	Discarded	-
60.	E060	-ve		-ve	Passed	-ve
61.	K011	-ve		-ve	Passed	-ve
62.	K012	-ve		-ve	Passed	-ve
63.	K013	-ve		-ve	Passed	-ve
64.	K014	+ve		+ve	Discarded	-
65.	K015	+ve		+ve	Discarded	-
66.	K016	+ve		+ve	Discarded	-
67.	K017	+ve		+ve	Discarded	-
68.	K018	-ve		-ve	Passed	-ve
69.	K019	-ve		-ve	Passed	-ve
70.	K020	+ve		+ve	Discarded	-
71.	K021	-ve		-ve	Passed	-ve
72.	K022	-ve		-ve	Passed	-ve
73.	K023	+ve		+ve	Discarded	-
74.	K024	-ve		-ve	Passed	-ve
75.	K025	-ve		-ve	Passed	-ve
76.	K026	-ve		-ve	Passed	-ve
77.	K027	+ve		+ve	Discarded	-
78.	K028	+ve		+ve	Discarded	-
79.	K029	-ve		-ve	Passed	-ve
80.	K030	-ve		-ve	Passed	-ve
81.	K031	-ve		-ve	Passed	-ve
82.	K032	+ve		+ve	Discarded	-
83.	K033	-ve		-ve	Passed	-ve
84.	K034	-ve		-ve	Passed	-ve
85.	K035	+ve		+ve	Discarded	-
86.	K036	-ve		-ve	Passed	-ve
87.	K037	-ve		-ve	Passed	-ve
88.	K038	+ve		+ve	Discarded	-
89.	K039	+ve		+ve	Discarded	-
90.	K030	+ve		+ve	Discarded	-
**Total**	**56/90 isolates were passed for the next step, whereas, 34/90 isolates were discarded**					

This table shows the isolation of lactic acid bacteria from Ethiopian fermented foods. This is copied from the excel
